# The potential effect of metformin on cognitive and other symptom dimensions in patients with schizophrenia and antipsychotic-induced weight gain: a systematic review, meta-analysis, and meta-regression

**DOI:** 10.3389/fpsyt.2023.1215807

**Published:** 2023-07-12

**Authors:** Vera Battini, Giovanna Cirnigliaro, Rodolfo Leuzzi, Eleonora Rissotto, Giulia Mosini, Beatrice Benatti, Marco Pozzi, Maria Nobile, Sonia Radice, Carla Carnovale, Bernardo Dell’Osso, Emilio Clementi

**Affiliations:** ^1^Department of Biomedical and Clinical Sciences, Pharmacovigilance & Clinical Research, International Centre for Pesticides and Health Risk Prevention, ASST Fatebenefratelli-Sacco, Università degli Studi di Milano, Milan, Italy; ^2^Department of Biomedical and Clinical Sciences, Psychiatry Unit 2, ASST Fatebenefratelli-Sacco, Università degli Studi di Milano, Milan, Italy; ^3^CRC “Aldo Ravelli” for Neurotechnology & Experimental Brain Therapeutics, Università degli Studi di Milano, Milan, Italy; ^4^Scientific Institute, IRCCS E. Medea, Bosisio Parini, Italy; ^5^Department of Psychiatry and Behavioral Sciences, Bipolar Disorders Clinic, Stanford Medical School, Stanford University, Stanford, CA, United States; ^6^Centro per lo studio dei meccanismi molecolari alla base delle patologie neuro-psico-geriatriche, Università degli Studi di Milano, Milan, Italy

**Keywords:** metformin, schizophrenia, meta-analysis, cognitive disorders, hypoglycemic drugs

## Abstract

**Introduction:**

Metformin has shown good efficacy in the management of antipsychotic-induced metabolic syndrome (MetS) in patients with schizophrenia or schizoaffective disorders. Its ability to induce antidepressant behavioural effects and improve cognitive functions has also been investigated: yet information has not been systematized. The aim of this study was therefore to investigate the effects of metformin on cognitive and other symptom dimension in schizophrenic patients treated with antipsychotics through a systematic review and meta-analysis.

**Methods:**

We searched PubMed, ClinicalTrials.Gov, Embase, PsycINFO, and WHO ICTRP database up to February 2022, Randomised Controlled Trials (RCT) evaluating patients diagnosed with schizophrenia and related disorders, who were treated with metformin as add-on therapy to antipsychotics for the treatment of weight gain and in which changes in psychiatric symptoms and cognitive functions were evaluated.

**Results:**

A total of 19 RCTs met the inclusion criteria. Meta-analysis was performed on 12 eligible studies. We found a positive trend after 24 weeks of treatment in schizophrenic patients with stable conditions [SMD (95%CI) = -0.40 (−0.82;0.01), OR (95%CI) = 0.5 (−2.4;3.4)]. Better performance was detected in the Brief Assessment of Cognition in Schizophrenia and Positive and Negative Syndrome Scale (PANSS) with low heterogeneity among studies. One study reported changes in BACS-verbal memory subdomain in favour of placebo [MD (95%CI) = -16.03 (-23.65;8.42)]. Gastrointestinal disorders, xerostomia, and extrapyramidal syndrome were the most reported adverse effects. Psychiatric adverse events were also described: in particular, symptoms attributable to a relapse of schizophrenia.

**Conclusion:**

Some degree of efficacy was found for Metformin in improving cognitive and other symptom dimensions in patients with Schizophrenia. Given the clinical relevance of this potential pharmacological effect, longer specific studies using adequate psychometric scales are strongly recommended. Likewise, how metformin acts in this context needs to be evaluated in order to enhance its efficacy or find more efficacious drugs.

## 1. Introduction

Schizophrenia (SCZ) is a chronic disorder characterized by a combination of psychotic symptoms (i.e., hallucinations, delusions, and disorganization) and motivational and cognitive dysfunctions. It affects about 1% of the world’s population and it is considered a high-cost disease due to the lifelong clinical course and the need of healthcare resource utilization (1).

Patients with SCZ have a mortality rate 2.6 times higher than that in the general population, mostly due to the occurrence of cardiovascular diseases and metabolic syndrome (MetS). The latter disease frequently arises in patients with SCZ due to a dysregulated and unhealthy lifestyle (2, 3), but it is also related to the treatment with second-generation antipsychotics (SGAs) (4–6). Considering pharmacodynamic implications, occupancies of H1 histaminergic and M1/M3 cholinergic receptors represent risk factors for increased levels of total cholesterol, HDL, LDL, insulin, and triglycerides (7, 8). Therefore, metabolic adverse effects of SGAs contribute to long-term risk of mortality and to short-term risk of obesity and MetS (9, 10). Current therapeutic options for weight control consist in dietary support and regular exercise, which, however, may not be sufficient for antipsychotic induced MetS (11, 12).

Eighty% of schizophrenic patients are also affected by cognitive alterations (13). Unfortunately, available antipsychotic drugs are not only ineffective on cognitive impairment (14), but can also worsen it (15, 16). Moreover, pharmacological cognitive enhancers in SCZ have limited efficacy and tolerability issues (17–19). Cognitive remediation (CR), a behavioural training–based intervention (20, 21), is currently the best option to improve cognition (22, 23), but it was proved to be ineffective in patients with Mets (24, 25).

In order to prevent MetS in psychiatric patients, many drugs have been evaluated, especially among antidiabetic drugs, that showed efficacy, good tolerability and compliance (26). One of the most studied drugs for preventing and treating antipsychotic-induced weight gain and MetS is metformin, a biguanide drug used to treat DM2 because of its high efficacy in lowering plasma glucose levels. It also exerts additional metabolic effects such as weight loss, reduction of triglycerides and LDL levels while increasing HDL and sensitivity to insulin (27). There is evidence that metformin can control antipsychotic induced MetS in schizophrenic patients (28). Of importance, its ability to induce antidepressant behavioural effects and improve cognitive functions has also been investigated (29). According to recent preclinical and clinical findings, metformin can penetrate through the Blood–Brain Barrier (BBB) into the central nervous system (CNS) where it promotes neuroprotective, neurotrophic, neurogenetic and anti-inflammatory effects (30). Furthermore, metformin reduces the inflammatory markers p-IKB, IL-1, and VEGF in neuronal cells reducing the neuroinflammation, a driver for neurotoxicity and the development of neuropsychiatric diseases (31, 32). A recent review concludes that metformin may activate the AMP Protein Kinase (AMPK), an enzyme that regulates the metabolic process of lipids and carbohydrates, leading to potential cognitive properties (33). Consistent with this hypothesis, many studies conducted in murine models have demonstrated the potential positive effect of metformin on cognition in neurodegenerative disorders (34) as diverse as Alzheimer’s disease (35), and traumatic brain injuries (36). At the same time, animal studies have focused on the beneficial effects of metformin on the CNS even across different neuropsychiatric conditions, such as anxiety (37), depression (38), schizophrenia-like symptoms (39) and seizures (40).

In a population-based longitudinal cohort study of diabetic individuals, participants using metformin showed higher performances in neuropsychological tests involving cognitive functions, especially verbal learning, working memory and executive function; even after adjusting for behavioural lifestyle or clinical conditions, these results did not change (41). In diabetic patients metformin had a neuroprotective function for the prevention of dementia (42). An improvement in cognitive function through the use of metformin was observed also in Huntington’s disease (43) and in a small sample of patients diagnosed with Fragile X Syndrome (44). In a randomised controlled trial, treatment with metformin showed anti-depressive effects in depressive and diabetic patients (45). Other clinical trials and observational studies, however, did not confirm the efficacy of metformin on cognitive function or on prevention against any form of dementia (46); moreover, metformin monotherapy has also been found to have negative effects in diabetic patients increasing the risk of Parkinson disease (47).

Even though data from both preclinical and clinical studies on possible pro-cognitive effects of metformin provide contrasting outcomes no attempts have been done to date to systematize and weight the available knowledge. We have thus specifically investigated the effects of metformin on psychiatric and cognitive functions through a systematic review of literature and meta-analysis of clinical trials in a selected population composed by schizophrenic patients treated with antipsychotics.

## 2. Materials and methods

### 2.1. Literature search

We followed the Preferred Reporting Items for Systematic Reviews and Meta-Analyses (PRISMA) guidelines (48). We submitted our Protocol at the International Prospective Register of Ongoing Systematic Reviews (CRD42021250690). We searched up to February 2022 PubMed, ClinicalTrials.Gov, Embase, PsycINFO, WHO ICTRP database using a search string containing two sets of words referring to 1) schizophrenic patients and 2) metformin that were combined using the Boolean operator “AND.” There was no language, date, document type, or publication status limitations for inclusion of records. Additional articles were collected through the reference lists of reviews and eligible studies we found. We did not plan to contact authors for unpublished data. An example of a search string is fully described in the Supplementary Material S1.

We included only Randomised Controlled Trials (RCT) evaluating people diagnosed with SCZ and related disorders (such as schizoaffective disorder, schizophreniform disorder, and delusional disorder) who were treated with metformin as add-on therapy to antipsychotics. We did not use any criteria for age, nationality or sex of the participants, duration/stage of illness, treatment setting, current clinical state, or symptom clusters. We considered metformin compared to placebo or other types of pharmacological interventions for the treatment of weight gain. We considered behavioural interventions only when combined with a pharmacological intervention. Primary outcomes were the changes in psychiatric and cognitive scales: the psychometric properties of the measuring instrument should have been validated and the measuring instrument should have not been modified for that trial.

### 2.2. Data extraction and processing

All titles and abstracts were assessed independently by two authors (GM, RL) to identify potentially relevant articles. Studies fulfilling the eligibility criteria were included and their full texts were retrieved and reviewed in duplicate (GM, ER). Discrepancies during the check of the two-step independent screening were resolved through the discussion with a third author (VB). Data were extracted by two researchers (RL, VB) and disagreements were resolved by consensus and consultation with the expert group (MP, CC, GC).

For every study the following data were extracted: First author; Year; Study duration; Study type (blinding/design); Number of subjects; diagnosis; number of males; age; antipsychotic(s) used and dose; control/comparator/placebo group; concomitant drugs; additional behavioural interventions; all outcomes of interest; Adverse Drug Reactions. Endpoint data were mainly chosen, mean change data if the former was not available.

### 2.3. Risk of bias assessment

This study was designed as an Intention-To-Treat (ITT) analysis. The risk of bias of included studies was assessed by three authors (GM, RL, ER) by using the Cochrane risk-of-bias tool for randomised trials (RoB 2) (49). Disagreements were resolved by consensus among them and a further consultation with the expert group (CC and VB). We planned to conduct a sensitivity analysis excluding studies rated with a high risk of bias if the number of remaining studies exceeds three.

### 2.4. Meta-analysis

A meta-analysis was performed by using the generic inverse variance method with a random effect model combining psychiatric scales reported by each study, which were Brief Psychiatric Rating Scale (BPRS) (50), Positive and Negative Syndrome Scale (PANSS) (51), Clinical Global Impression Scale (CGI) (52), Global Assessment of Functioning (GAF) (53), Scale for the Assessment of Negative Symptoms (SANS) and Scale for the Assessment of Positive Symptoms (SAPS) (54), Brief Assessment of Cognition in SCZ (BACS) (55) and Patient Health Questionnaire-9 (PHQ9) (56). Among scales, when more than one tool was available, a priority order was defined considering their impact on cognitive assessment. The procedure is clearly described in Supplementary Material S2. Thus, the final priority order adopted was as follows: BACS composite T score > BACS verbal memory T score > PANSS> BPRS > GAF > CGI > SANS and SAPS > PHQ9.

Assumptions were made regarding missing SDs using data of similar studies in terms of population, number of patients, and the point estimate. Forest plots were created for the main outcomes. Sensitivity analyses were performed excluding these studies to check their influence in the results.

In order to help the reader in the interpretation of results we also provided Mean Differences (MDs) of subgroup analyses concerning single scales. In these analyses, we included all studies reporting results of the scale of interest, we did not follow the priority order reported above.

RevMan 5 was the chosen tool to perform the meta-analysis (57).

### 2.5. Meta-regression

It is known that the amount of adipose tissue of the patients impacts on the pharmacokinetic properties of antipsychotics and thus on their therapeutic effect (58–61).

We then explored the influence of the Body Mass Index (BMI) at baseline on the treatment effect (SMD) of our main meta-analysis to evaluate if a better response is more related to a better response to the psychiatric treatment than the efficacy of metformin in the regulation of psychiatric and cognitive symptoms. A random-effects meta-regression model with Knapp-Hartung method was performed. Data are provided by a regression bubble plot. The [meta] R package was used to perform meta-regression (62).

### 2.6. Post-hoc analysis

As specified in our protocol, we planned to check for eventual useful analyses that were not previously considered. We therefore decided to perform a sub-group analysis of studies reporting data at 12 and 24 weeks.

Since six studies reported an additional dietary and physical exercise control to patients, we also investigated the role of Lifestyle interventions in changes of scales with a sensitivity analysis.

### 2.7. Assessment of heterogeneity

We interpreted I*^2^* estimate greater than or equal to 50% together with a statistically significant Chi*^2^* statistic as evidence of substantial heterogeneity. We also visually inspected graphs to investigate the possibility of statistical heterogeneity and discussed it in the proper section.

### 2.8. Differences between protocol and review

We clearly state down below the deviations from the original protocol registered in PROSPERO:

- We included those studies in which less than 10% of patients were diagnosed with bipolar disorder.
- Our aim is to whether metformin as add-on therapy improved or not SCZ symptoms, with a particular interest in cognitive functions. Therefore, within psychiatric scales, when more than one tool was available in the study, we applied a priority order as described in the statistical analysis section. This was due to the presence of more than one outcome in several studies.
- We changed the statistical method of the meta-analysis due to the type of outcomes reported in the literature. We preferred the generic invariance methods instead of the Mantel–Haenszel because data were all reported in continuous variables (endpoint data or mean changes) and it wasn’t possible to convert them into dichotomous. However, we decided to provide Odds ratios (OR) by converting the results of total Standardized Mean Differences (SMDs) with the Hasselbach & Hedges’ method (63) in order to help the reader in the interpretation of our results:

$$\mathrm{lnOR}={\mathrm{SMD}}^{*}1.81\to \mathrm{OR}=e^{\mathrm{SMD}*1.81}.$$

$${\mathrm{SE}}_{\mathrm{lnOR}}={\mathrm{SE}}_{\mathrm{SMD}}{}^{*}1.81$$

- We provided sub-group analyses for each scale.
- Due to the paucity of studies retrieved, we could not perform neither funnel plots, nor analyses on the effects of high risk of bias and the role of diabetes.

## 3. Results

### 3.1. Literature search

**Figure 1:**
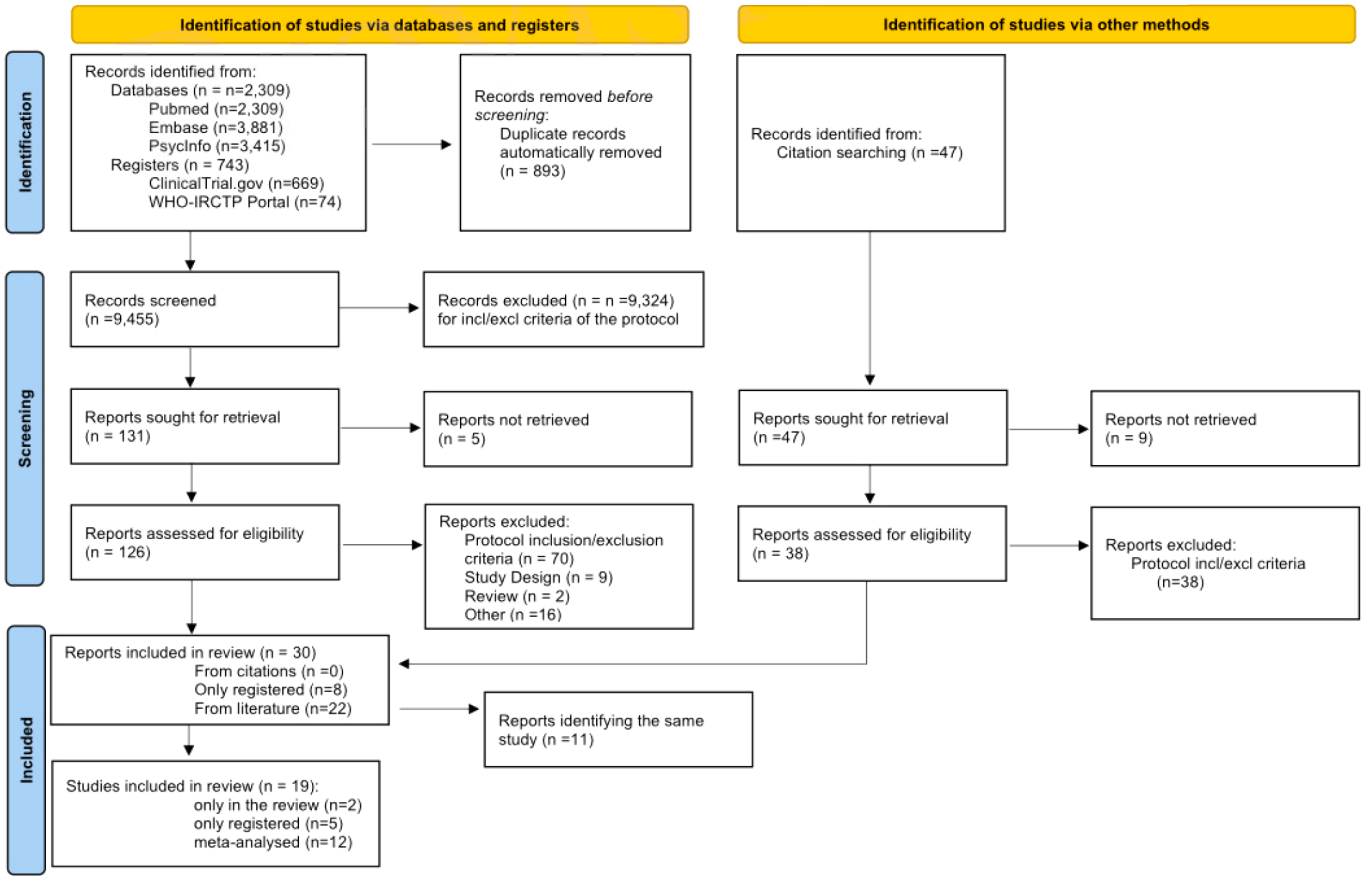
The PRISMA 2020 flowchart.

The study selection and screening process is presented in the PRISMA 2020 flowchart (Figure 1). The electronic search identified 9,605 records from literature databases and 743 trials in study registers. After duplicates removal, 9,455 records were screened. Thirty-eight records were retrieved by manual search in the reference lists of relevant reviews and included studies for full-text analysis. Nine-teen studies eventually met our eligibility criteria and were included in the review: 12 were eligible to perform meta-analysis, 2 reported only qualitative data, and 5 were only present in trial registers without any result. These latter trials will be described in a separate section.

### 3.2. Characteristics of the included studies (*n* = 14)

Full description of trials is available in Table 1 and Supplementary Material S1. All studies were designed as comparison between metformin and placebo, except two: Mondal et al. (64) that added one arm treated with topiramate, and Wu et al. (65) that explored the influence of lifestyle interventions. The duration of the included studies was between 12 weeks and 36 weeks. The prescribed dosage of metformin varied from 500 mg/die to 2000 mg/die. All studies included adult patients who were less than 80 years, diagnosed of SCZ (DSM-IV) and under stable treatment with antipsychotics. Four studies (66–69) included patients diagnosed with bipolar disorder and other psychiatric disorders (DSM-IV), however in a negligible percentage: as an example, Agarwal et al. 2 patients out of 30; Baptista et al. 4 patients out of 80. In 5 trials (66, 70–73) only overweight/obese patients were included, and 6 trials reported diabetes or prediabetes among the exclusion criteria (66, 69, 70, 72, 74, 75). All studies, except Mondal et al. (64), reported any other chronic disease (such as thyroid, liver or renal dysfunction, cardiovascular disease) and pregnancy among the exclusion criteria. The mean age at diagnosis was between 21 and 26 years. The disease duration varied from months (first episode) to more than two decades. Clozapine, olanzapine, aripiprazole, and risperidone were the most used antipsychotics. In 3 trials the concomitant use of mood stabilizers, benzodiazepines and antidepressants was permitted (65, 69, 75). Lifestyle intervention involving diet and physical exercise was provided in 6 trials (65, 66, 68, 72, 76, 77). All studies included validated psychometric scales for the clinical assessment; PANSS (65, 70, 72–74, 77) and BPRS (66–69, 71, 76) were the most used.

**Table 1:** Characteristics of the included studies.

First Author and Publication Date	Trial registration number	B	Duration (weeks)	Inclusion criteria	Exclusion criteria	Arms	Scales
Agarwal 2021	NCT02167620	Y	16	Age 17-45 BMI > 25 kg/m2 Schizophrenia or schizoaffective disorder or BD Prediabetes or T2DM Stable treatments with Antipsychotics	Comorbid psychiatric disorders T1DM Liver or renal dysfunction Substance abuse A_1_C > 9.5%, or symptomatic hyperglycaemia with metabolic decompensation Reported lack of tolerability/efficacy for metformin Hyperglycaemic or lipid-lowering medications Pregnancy	Metformin vs. Placebo	CGI GAF BPRS BACS
Baptista 2006	–	Y	14	Clinically stable inpatients. Severe schizophrenia or schizoaffective disorder.	Any other chronic diseases. Hormone replacement therapy.	Metformin vs. Placebo	BPRS
Batista 2007	–	Y	12	Age > 18 yrs stable treatment with olanzapine Any mental disorders Normal physical and laboratory tests	Any other chronic diseases Hormone replacement therapy	Metformin vs. Placebo	BPRS
Carrizo 2009	–	Y	14	Age > 18 yrs Stable treatment with olanzapine Normal physical and laboratory tests	Hormone replacement therapy	Metformin vs. Placebo	BPRS
Chen 2013	NCT013006637	Y	24	Age 20-65 yrs BMI ≥24 kg/m^2^ schizophrenia or schizoaffective disorder (DSM-IV) Stable treatment with clozapine Metabolic syndrome	T1DM or T2DM Hyperglycaemic or lipid-lowering medications FPG levels ≥126 mg/dL Reported lack of tolerability/efficacy for metformin Pregnancy	Metformin vs. Placebo	PANSS
Chiu 2016	NCT02751307	Y	12	Age 20-65 yrs Schizophrenia or schizoaffective disorder (DSM-IV) Stable treatment with clozapine Metabolic syndrome	T1DM or T2DM Hyperglycaemic or lipid-lowering medications FPG levels ≥126 mg/dL Reported lack of tolerability/efficacy for metformin Pregnancy	Metformin (500 mg) vs. Metformin (1 g) vs. Placebo	PANSS
Hebrani 2015	–	Y	20	Age 18-75 yrs BMI >25 kg/m2 Inpatients Schizophrenia Stable treatment with clozapine	Hormone replacement therapy Other serious medical or mental illness Any other chronic diseases Substance abuse Discharge from the hospital by patient’s own consent Refusal to complete the study and follow-up Pregnancy or breastfeeding	Metformin vs. Placebo	BPRS
Mondal 2014	–	–	24	Schizophrenia.	–	Metformin vs. Topiramate vs. Control	SAPS SANS
Siskind 2021	ACTRN12617001547336	Y	24	Age 18-64 yrs 18 ≤ BMI ≤ 40 kg/m^2^ Schizophrenia or Schizoaffective disorder (DSM-IV) Clozapine <2 weeks FastingBG ≤6.0 mmol/L	T1DM or T2DM Reported lack of tolerability/efficacy for metformin Hypoglycaemic agents Weight-loss medications Obesity induced by other endocrinologic disorder (e.g., Cushing’s Syndrome, Hypothyroidism) Corticosteroids or other hormone therapy (except estrogens or thyroxine) > 10 days |CKD (eGFR<60 mL/min) Previous obesity-related surgical treatment Any unstable medical illnesses Pregnancy or breastfeeding	Metformin vs. Placebo	PANSS GAF
Tang 2021	–	Y	36	Age 16-40 yrs First-episode psychiatric disorders (DSM-IV) CGI-S ≤ 3 ≥5% of weight gain with AP treatment	BMI < 18.5 kg/m^2^ T1DM or T2DM Thyroid, liver or renal dysfunction Cardiovascular disease Non-naive users for metformin Intellectual disability Substance abuse Hypoglycaemic agents Weight-loss medications Any unstable medical illnesses Pregnancy or breastfeeding	Metformin vs. Placebo	BPRS GAF PHQ-9
Wang 2012	–	Y	12	Age 18-60 yrs BMI > 25 kg/m2 First-episode of schizophrenia (DSM-IV) PANSS≤60 >3 months under the same AP >7% of weight gain with one year of AP treatment.	Other psychiatric diagnoses Other clinical conditions Pregnancy or breastfeeding	Metformin vs. Placebo	PANSS
Wu 2008a	NCT00451399	Y	12	Age 18-45 yrs Outpatients First-episode of schizophrenia (DSM-IV) PANSS≤60 Duration of illness <12 months Caregivers required Stable treatments with Antipsychotics >10% of weight gain with one year of AP treatment	Any other psychiatric diagnoses. Any other clinical conditions Dietary restriction Substance abuse. Pregnancy or breastfeeding.	Metformin vs. Placebo vs. Metformin (+Life style) Placebo (+Life style)	PANSS
Wu 2008b	–	Y	12	Age 18-50 yrs Inpatients First-episode of schizophrenia (DSM-IV) No APs/recreational drugs for at least 3 months	Other clinical conditions Pregnancy or breastfeeding	Metformin vs. Placebo	SAPS SANS
Wu 2016	NCT01778244 NCT01206153	Y	24	*NCT01778244* Age 18-40 yrs Schizophrenia (DSM-IV) Dyslipidaemia within the first year of AP treatment Duration of illness <12 months only one AP in the last 3 months PANSS ≤ 60 Caregiver required	Any other psychiatric diagnoses Liver or renal diseases Cardiovascular disease T1DM or T2DM Pregnancy or breastfeeding.	Metformin vs. Placebo	PANSS
				*NCT01206153* Age 18-40 yrs Outpatients Schizophrenia (DSM-IV) Amenorrhea > three months Duration of illness <12 months Only one AP in the last 6 months, with no more than a 25% change in dosage. PANSS ≤ 60 Caregiver required.			

B, Blindness; BACS, Brief Assessment of Cognition in Schizophrenia; BPRS, Brief Psychiatric Rating Scale; BD, Bipolar Disorder; BMI, Body Mass Index; CGI, Clinical Global Impression Scale; DSM-IV, Diagnostic and Statistical Manual of mental disorders, fourth version; GAF, Global Assessment of Functioning; FPG, fasting plasma glucose; PANSS, Positive and Negative Syndrome Scale; PHQ9, Patient Health Questionnaire-9; SANS, Scale for the Assessment of Negative Symptoms; SAP, Scale for the Assessment of Positive Symptoms; T1DM, Type 1 Diabetes Mellitus; T2DM, Type 2 Diabetes Mellitus; Y, yes; yrs, years.

### 3.3. Scales

The following numerical results must be read as “*metformin compared to placebo*.”

**Figure 2:**
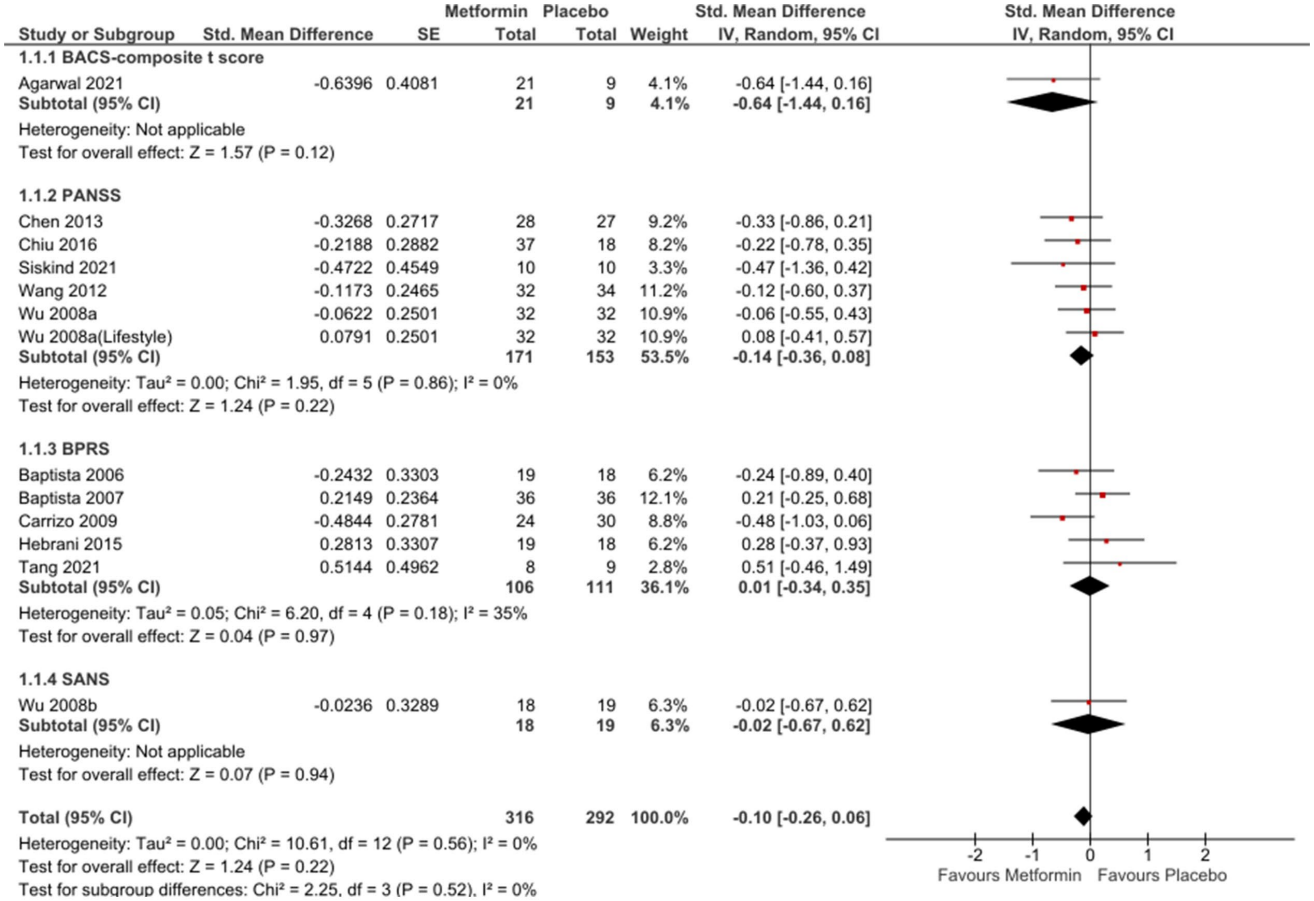
Metformin compared to Placebo considering all scales.

In the analysis of 12 studies (65–74, 76, 77), metformin resulted in a favorable position against placebo (Figure 2), even if not statistically significant [SMD (95% CI) = −0.10 (−0.26; 0.06), OR (95% CI) = 0.8 (−1.4; 3.1)]. No significant differences were seen when studies with missing SDs or those with lifestyle intervention were excluded [SMD (95% CI) = −0.09(−0.27; 0.09), OR (95% CI) = 0.9 (−1.5;3.16) and SMD (95% CI) = −0.02(−0.22; 0.19), OR (95%CI) =1.0 (−1.4; 3.3), respectively].

**Figure 3:**
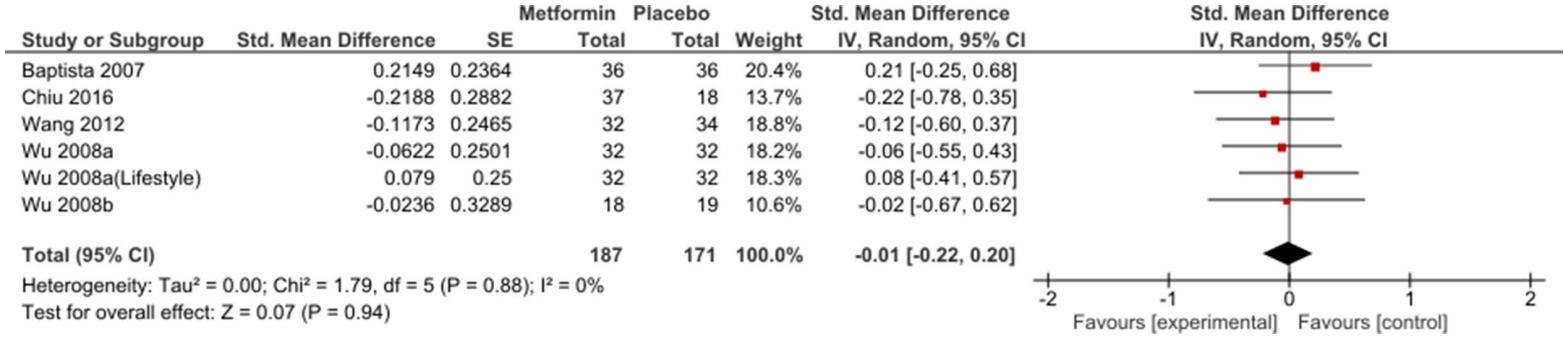
Sub-group analysis at twelve weeks.

**Figure 4:**
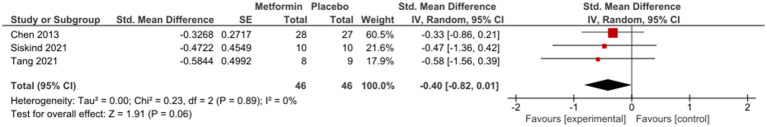
Sub-group analysis at twenty-four weeks.

Sub-group analysis examining studies with same duration of follow-up found non-significant results at 12 weeks [SMD (95%CI) = −0.01(−0.22; 0.20), OR (95% CI) = 1.0 (−1.4; 3.4)] (Figure 3); however, a subsequent improvement at 24 weeks [SMD (95% CI) = −0.40 (−0.82; 0.01), OR (95% CI) = 0.5 (−2.4; 3.4)] (Figure 4). No significant differences were seen when studies with missing SDs or those with lifestyle intervention were excluded.

Forest plots of single-scale analyses of PANSS, BPRS and GAF are available in Supplementary Material (Figure S1-S3 respectively). Better performances were detected by BACS-composite t-score [MD (95%CI) = 1.26 (−0.42; 2.94)], result from one study (66) and PANSS [MD (95% CI) = −2.26 (−5.90; 1.39)], result from 5 studies (65, 70, 72–74), compared to BPRS [MD (95% CI) = −0.57 (−2.56; 1.41)], result from 6 studies (66–69, 71, 76). One study (66) reported changes in BACS-verbal memory in favour of placebo [MD (95% CI) = −16.03 (−23.65; 8.42)]; on the other hand, another study (77) described non-significant results related to SANS [MD (95% CI) = −0.05 (−1.38; 1.28)] and SAPS [MD (95%C I) = 0.09 (−0.67; 0.85)] and similar results were described by Mondal et al. (64) Three studies (66, 69, 72) reported a not significant improvement in GAF [MD (95% CI) = 0.35 (−2.51; 3.21)] and PHQ9 [MD (95% CI) = −2.50 (−1.70; 2.07)], only one study (69).

### 3.4. Metformin vs. topiramate

Only one study (64) compared metformin to another drug used to control the increase of weight in schizophraenic patients. The Authors did not report any quantitative result, they only state that no differences were found in SAPS and SANS scales among groups after 24 weeks.

### 3.5. The influence of BMI at baseline

**Figure 5:**
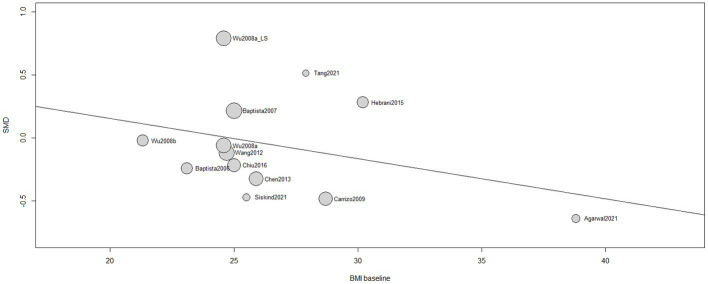
The influence of BMI at baseline, meta-regression.

A non-significant influence of BMI at baseline in the treatment response [*β* (95%CI) = −0.0320 (−0.0982;0.0343), *I*^2^ = 45.05%, *R*^2^ = 4.60%, test of moderators: *F* = 1.1279; *p* = 0.3110] was found (Figure 5). Same results were reported by 5 single studies (66, 67, 75–77).

### 3.6. Adverse events

The general adverse events that were reported by authors were related to gastrointestinal discomfort, xerostomia, and extrapyramidal syndrome. Several psychiatric adverse events were also described, particularly some symptoms attributable to a relapse of SCZ (psychotic relapse/exacerbation, unstable/worsening of illness), others to mood alteration (depression, suicidality, irritated/bad mood), and finally some unspecific symptoms such as insomnia and agitation (Supplementary Table S2).

### 3.7. Studies in trial registers (*n* = 5)

Five trials were registered on clinicaltrial.gov (78). NCT01654640 was terminated because they were not able to recruit enough patients; in NCT02140788, the Principal Investigator left the Institution, and the trial was interrupted. NCT03271866, reported as “unknown status,” focuses on the effect of metformin on cognitive impairment. NCT03708549 is a phase 4 trial that is still recruiting; the aim of the study is to compare berberine and metformin and the evaluation of the PANSS is among the secondary outcomes. NCT04865835 is a phase I trial that has been completed and it is likely under review; however, the aim of this study is to evaluate in pharmacokinetic of a novel substance compared to metformin, which is not specifically our outcome of interest.

### 3.8. Risk of bias

**Figure 6:**
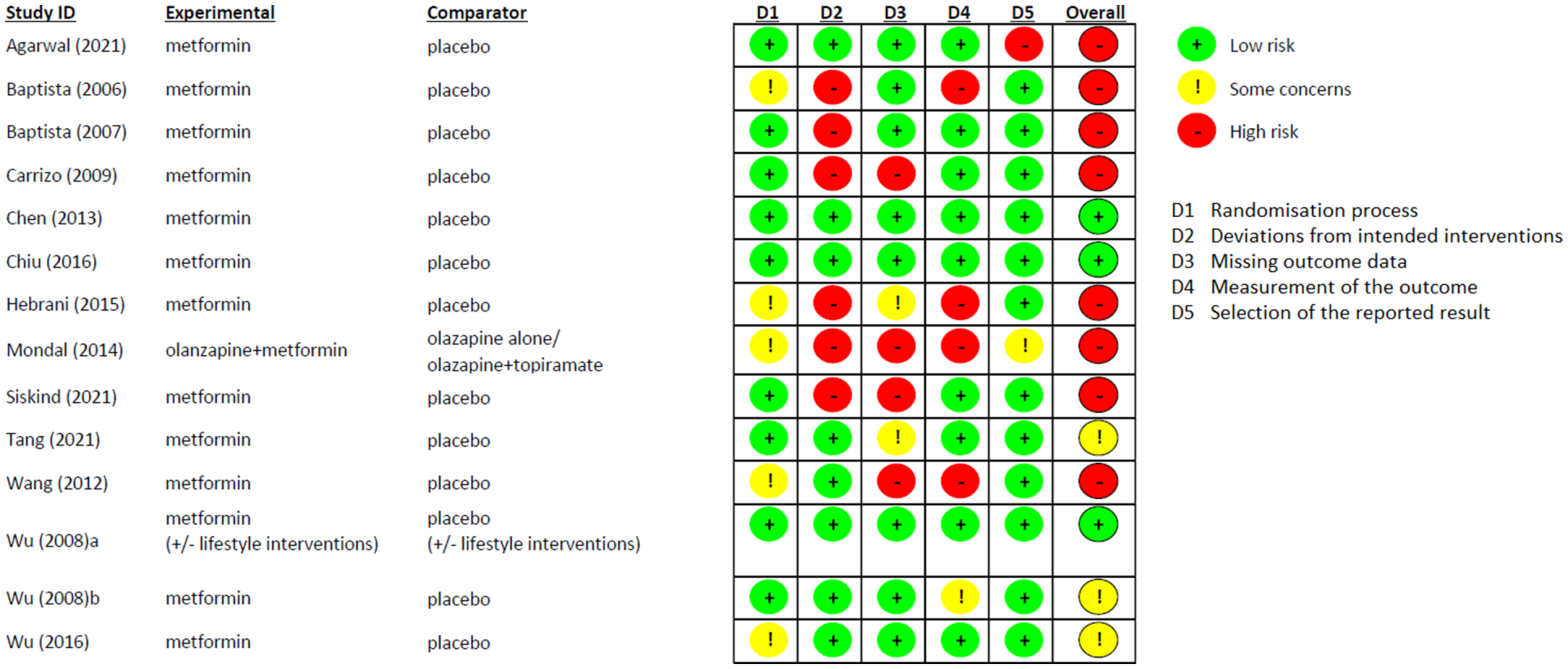
Risk of Bias of the included studies.

Risk of Bias of the included studies is shown in Figure 6. In general, most of the studies reported high risk of bias (8/14). However, the randomisation process was favorably assessed in all studies and the “Deviations from intended interventions” domain was the one that highly influenced the general results because of the Per Protocol analysis used in 6 studies (64, 67, 68, 71, 72, 76). Three studies were considered with low-risk of bias in all domains (65, 70, 74), and only one study was at real high-risk of bias, since none of the fields were assessed without any concern (64).

## 4. Discussion

Since its approval in 1958, metformin has become one of the most widely used therapy for DM2 and it still represents the first-line therapy. While improved mitochondrial metabolism and insulin signaling are generally suggested as mechanisms underlying beneficial pro-cognitive effects of antidiabetic drugs, other factors such as active adenosine 5′- monophosphate-activated protein kinase (AMPK) activation, modulation of microglial phenotype, mTOR inhibition, and increased autophagy in the brain might be involved (79). Because of these multiple mechanisms, many studies have already described potential effects of metformin in treating conditions other than diabetes (80–83); here we assessed for the first time through a systematic review the potential effects of metformin on cognitive functions and psychiatric symptoms in schizophrenic patients treated with antipsychotics.

A general positive trend was seen after 24 weeks of treatment [SMD (95%CI) = −0.40 (−0.82;0.01), OR (95% CI) = 0.5 (−2.4; 3.4)] in patients who were generally considered in stable conditions.

Unfortunately, the relatively short period of investigation of the included studies (only one study up to 36 weeks) could mask the neuroprotective effects of metformin since in previous RCTs they seemed to emerge after long-term use (6–8 years) (42). A better improvement was related to those scales allocated in the higher positions of our priority scale (BACS composite T score > BACS verbal memory T score > PANSS) with low heterogeneity among studies, then worsening while going further with the other psychiatric tools (BPRS > GAF > CGI > SANS and SAPS > PHQ9). Furthermore, among all cognitive domains assessed by the BACS (verbal memory, working memory, motor speed, attention, executive functions, and verbal fluency), only verbal memory was in favour of placebo [MD (95%CI) = −16.03 (−23.65; 8.42)] (66). This finding could indicate a greater influence of metformin on cognitive rather than psychiatric symptoms, but it is not possible to draw any conclusions since only one study (66) reported results for the BACS composite t-score [MD (95% CI) = 1.26 (−0.42; 2.94)]. This scale is specifically designed for the evaluation of cognitive functions, but the small sample size of this trial could be an important limit for the power of the performed analysis. It is interesting to note that we were able to retrieve another trial that was registered in 2017 (84): the aim of this study was to investigate the impact of metformin on cognitive impairment in schizophrenic patients with or without MetS. This 24-week trial should recruit 80 patients and compare metformin group versus controls on PANSS Scale, Calgary Depression Scale for SCZ (Chinese version) and MATRICS Consensus Cognitive Battery. Unfortunately, the last version of the protocol was submitted in 2020 and the recruiting status is unknown. We could not therefore include their findings in our analysis. However, some indirect clinical evidence on a potential enhancement of cognitive function may come from neurodegenerative disorders: metformin has shown potential therapeutic benefit against mild cognitive impairment and Alzheimer’s disease among diabetic patients (85), even if the use of metformin for prevention of dementia in older non-diabetic adults is not currently recommended (42).

No correlation was seen with the patients’ BMI at baseline, thus indicating no potential differences in the use of metformin in first-episode psychosis or under chronic treatment with SGAs. Literature findings report how the earlier the onset of SCZ and the longer its duration, the worse is the clinical response to antipsychotics (86). One of the hypotheses behind this evidence-based finding is that progressive brain tissue loss occurs in schizophrenic patients, and this neurobiological alteration would interfere with the effectiveness of metformin as much as antipsychotic therapy (87). Among the studies included in our analysis, only four used a first episode psychosis as an inclusion criterion (65, 69, 73, 77). Therefore, despite missing data, we can assume that most patients were enrolled after a duration of illness that could impact negatively on the efficacy of pharmacotherapy. Disease duration ranged from 6.8 months to 27.8 years and in seven studies it was not reported (64, 67–70, 72, 76). Further studies including disease onset and duration information or that include only first-episode patients are therefore recommended.

Regarding the antipsychotic drugs that were used in the included studies, all patients were mainly treated with SGAs, while only three studies (66, 69, 76) reported concurrent treatment with first-generation antipsychotics, confirming the known strong association between weight gain and SGAs (4). Among them, clozapine and olanzapine were responsible for the highest incidence of MetS, consistently with a recent network meta-analysis on glyco-metabolic adverse effects of antipsychotics (7).

As all the other drugs available on the market, metformin might cause adverse effects, although the most frequent ones are considered mild enough to recommend maintaining the use of metformin unless renal/hepatic function deterioration arises (88). Metformin doses that were used in all the included trials were in line with the latest recommendation (89) and no high-concerning adverse event was therefore reported. Gastrointestinal disorders were the most described events; this is not surprising as they are known to be very common at the start of the therapy and can be minimized by dose reduction, slower dose titration and after-meal administration (89). Physical symptoms, namely xerostomia, headache and extrapyramidal syndrome were also reported; this indicates that it is worth recommending caution and careful patients’ counseling before starting metformin, as adverse events may represent an additional risk factor for dropping out of the overall psychiatric treatment (90). Somehow unexpectedly, few psychiatric adverse events were reported, these were essentially from relapse of SCZ mood alteration, insomnia, and agitation. Based on the known mechanism of action of metformin a clear causal relationship between psychiatric symptoms and metformin appear improbable. Rather, it is likely that they arose due to the chronic course of the underlying psychiatric disease.

However, considering the observed adverse effects, it is important to assess the risk–benefit ratio of an add-on therapy with metformin. Metformin has proved its efficacy on cardiometabolic complications, which cause a three-times higher mortality risk in SCZ patients than that of the general population (91). When there is balance between the odds of therapeutic effects and the risk of adverse events, metformin administration in these patients seems beneficial, especially if metformin might exert improvements in pro-cognitive functions, which is of clinical relevance. However, such evidence is not yet solid enough and it is premature to propose a change in current clinical practice and in medical prescription at this stage. Further studies considering the benefit/risk ratio are warranted.

### 4.1. Strength and limitations

Chronic treatment with SGAs is essential in the control of psychotic symptoms and the prevention of relapses in SCZ. For this reason, it is widely adopted in clinical settings, even though it can increase the risk of MetS and negatively impact cognitive performance thus worsening the therapeutic compliance, already impaired by the pathology itself. Therefore, the identification of a treatment that can contrast dysmetabolism and cognitive impairment in psychiatric patients would have a high impact in psychiatric clinical practice. Not only has metformin previously shown to be effective in reducing MetS, but it is also considered a low-cost drug, with a well-known safety profile. Our primary aim was to verify the hypothesis, previously emerged from several preclinical and clinical studies, that metformin may exert pro-cognitive effects also in psychotic patients, with or without DM2. This meta-analysis, in addition to its clinical relevance, represents an original perspective in the current literature background.

The first obstacle in investigating our primary objective was that only one study (66) used a specific assessment instrument for cognitive function, the BACS. This is the most widely adopted and validated scale that assesses cognition’s domains most impaired and correlated with outcome of SCZ (55, 92). Unfortunately, it is still underused in clinical practice, while the clinical course and functioning of SCZ are usually assessed by several validated psychometric scales, the main ones being BPRS, PANSS, CGI, GAF. Most of these latter scales contain specific items concerning the patient’s cognitive asset. Thus, since partial scores of these items were not available in the analyzed studies, we applied the priority order described above, that is an original method in order not to neglect valuable information for our primary aim. However, further studies with appropriate scales are warranted.

Another limit of our analysis is the relatively short period of investigation (only one study up to 36 weeks) while neuroprotective effects of metformin observed in previous RCT seem to emerge after long-term use (8 and 6 years) (42). Only 3 RCTs were assessed with low risk of bias, and we could not perform any sensitive analysis excluding those with high risk. However, considering that our aim was defining changes in measurements, the most important domains for our results were the quality of the randomisation process (domain 1) and the measurement of the outcome (domain 4), which were both considered at low risk of bias in the 64% of the included studies.

## 5. Conclusion

Metformin has been previously shown to reduce weight gain and the risk of MetS in schizophrenic patients treated with SGA; our systematic review suggests that it may also improve psychiatric and cognitive symptoms in the same population. Given the clinical relevance of this potential pharmacological effect of metformin, longer specific studies exploring cognitive performance and using adequate psychometric scales are strongly recommended.

## Data availability statement

The raw data supporting the conclusions of this article will be made available by the authors, without undue reservation.

## Author contributions

VB, GC, RL, ER, GM, BB, MP, MN, SR, CC, BD’O, and EC contributed to the study conception and design. Material preparation and data collection were performed by VB, RL, GM, and ER. Data analyses were performed by VB, ER, and CC. The first draft of the manuscript was written by VB, RL, GC, and ER. All authors contributed to the article and approved the submitted version.

## Funding

This work was supported by Università degli Studi di Milano (Piano di Sostegno alla Ricerca, LINEA 3 to CC) which are gratefully acknowledged. The funding public institutions had no role in any part of the work.

## Conflict of interest

BD’O has received lecture honoraria that are not related to the work submitted for publication, from Angelini, Janssen, Lundbeck, Bromatech, Otzuka, and Neuraxpharm.

The remaining authors declare that the research was conducted in the absence of any commercial or financial relationships that could be construed as a potential conflict of interest.

## Publisher’s note

All claims expressed in this article are solely those of the authors and do not necessarily represent those of their affiliated organizations, or those of the publisher, the editors and the reviewers. Any product that may be evaluated in this article, or claim that may be made by its manufacturer, is not guaranteed or endorsed by the publisher.
