# Supplementary material for: The potential effect of metformin on cognitive and other symptom dimensions in patients with schizophrenia and antipsychotic-induced weight gain: a systematic review, meta-analysis, and meta-regression
Source: Front Psychiatry. 2023 Jul 12;14:1215807. doi: 10.3389/fpsyt.2023.1215807 (PMC10370497; doi:10.3389/fpsyt.2023.1215807)

Supplementary Material

The potential effect of metformin on cognitive and other symptom dimensions in patients with schizophrenia and antipsychotic-induced weight gain: a systematic review, meta-analysis, and meta-regression

**Vera Battini^1^*, Giovanna Cirnigliaro^2^, Rodolfo Leuzzi^2^, Eleonora Rissotto^2^, Giulia Mosini^1^, Beatrice Benatti^2,5,^ Marco Pozzi^3^, Maria Nobile^3^, Sonia Radice^1^, Carla Carnovale^1^, Bernardo Dell’Osso^2,4,5,6^, Emilio Clementi^1,3^**

*** Correspondence:**

Vera Battini, MPharm, Cand. PhD

Department of Biomedical and Clinical Sciences

Università degli Studi di Milano

Via G.B. Grassi 74

Milan 20157, Italy

E-mail: vera.battini@unimi.it

**Index:**

# Supplementary Material S1: Pubmed search string

Supplementary material S2: Creation of the scale priority order.

Table S1: Additional information of the included randomized controlled trials.

Table S2: Adverse Events (AEs) reported in every single trial

Figure S1: Metformin compared to Placebo considering PANSS

Figure S2: Metformin compared to Placebo considering BPRS

Figure S3: Metformin compared to Placebo considering GAF

# Supplementary Material S1. Pubmed search string

("Schizophrenia Spectrum and Other Psychotic Disorders"[Mesh] OR "Schizophrenia"[Mesh] OR Schizo* [tiab] OR Psych*[tiab])AND (“Pharmacological” [tiab] OR "Antipsychotic Agents"[Mesh] OR "Tranquilizing Agents"[Mesh] OR Antipsychotic*[TIAB] OR “Major Tranquilizers” [TIAB] OR “Tranquillizing Agents” [TIAB] OR “Major Tranquillizing Agents” [TIAB] OR Neuroleptic* [TIAB] OR Antipsychotic* [TIAB] OR Amisulpride[TIAB] OR Aripiprazole[TIAB] OR Asenapine[TIAB] OR Chlorpromazine[TIAB] OR Clozapine[TIAB] OR Droperidol[TIAB] OR Fluphenazine[TIAB] OR Haloperidol[TIAB] OR Iloperidone[TIAB] OR Loxapine[TIAB] OR Lurasidone[TIAB] OR Mesoridazine[TIAB] OR Molindone[TIAB] OR Olanzapine[TIAB] OR Paliperidone[TIAB] OR Periciazine[TIAB] OR Perphenazine[TIAB] OR Pimozide[TIAB] OR Promazine[TIAB] OR Quetiapine[TIAB] OR Risperidone[TIAB] OR Sertindole[TIAB] OR Sulpiride[TIAB] OR Thioridazine[TIAB] OR Trifluoperazine[TIAB] OR Ziprasidone[TIAB] OR Zotepine[TIAB] OR Zuclopenthixol[TIAB] OR levomepromazine [TIAB] OR promethazine [TIAB] OR tiapride[TIAB] OR clotiapine[TIAB] OR cariprazine [TIAB] OR brexpiprazole[TIAB]) AND ("Metformin"[Mesh] OR “Metformin” [tiab] OR "Hypoglycemic Agents" [Mesh] OR “Antidiabetic” [tiab] OR “Hypoglycemic” [tiab] OR “Weight control”[tiab] OR “weight loss”[tiab] OR “weight reduction” [tiab] OR “weight decrease” [tiab] OR “overweight”[tiab] OR “obese”[tiab] OR “obesity”[tiab] OR “antipsychotic-induced weight”[tiab])

**Supplementary material S2.** Creation of the scale priority order.

We calculated, where possible, the ratio between the number of items concerning cognition and the total number of items available in each scale. The following ratio were calculated for the scale here presented in alphabetic order.

- BACS: 5 items concerning cognition/5 total items=1
- BPRS: 2 items concerning cognition/18 total items=0,11
- CGI: no specific items on cognition
- GAF: no specific items on cognition
- PANSS: 6 items concerning cognition/30 total items=0,2
- PHQ-9: 1 item concerning cognition/9 total items=0,11
- SAPS and SANS: 3 items concerning cognition/59 total items=0,05

GAF and CGI scales lack specific items on cognition. GAF scale measures how much symptoms affect the patient's general functioning on a scale of 0 to 100 [1]. CGI scale is a seven-point scale in which the clinician must rate the severity of the patient's illness at the time of assessment [2]. Considering the extensive clinical use of these scales in patients with schizophrenia and the strong association between cognitive deficits, severity of illness, and low functioning in schizophrenia [3,4], we placed GAF and CGI scales in an intermediate position in relation to the others. SAPS and SANS scales were considered together since they are always performed in pairs. They also separately investigate two areas of schizophrenia (positive and negative symptoms) that in the PANSS are considered together and affect its total score [5,6]. The PHQ9 scale was used in only one study. We placed it at the bottom of our priority order since it is the only self-report scale, thus less reliable than the other clinical rating scales. Moreover, it was constructed to investigate symptoms of depression [7], so we consider its use in patients with schizophrenia somewhat improper.

We therefore created the following priority list:

1. BACS
2. PANSS
3. BPRS
4. GAF
5. CGI
6. SAPS and SANS
7. PHQ-9

References:

1. Hall R.C.W. Global assessment of functioning: a modified scale. Psychosomatics. 1995;36:267–275. doi: 10.1016/S0033-3182(95)71666-8.
2. Guy W: Clinical Global Impression. ECDEU Assessment Manual for Psychopharmacology, revised National Institute of Mental Health, Rockville, MD. 1976
3. Nasrallah HA, Smeltzer DJ. In: Contemporary diagnosis and management of the patient with schizophrenia (2nd Edition). Handbooks in Healthcare Co., Newton, Pennsylvania. 2011.
4. Alicia Ruiz-Toca et al. Social Cognition Mediates the Impact of Processing Speed and Sustained Attention on Global Functioning in Schizophrenia. PMID: 36695854 DOI: 10.7334/psicothema2022.8
5. Theo G M van Erp et al. Converting positive and negative symptom scores between PANSS and SAPS/SANS. Schizophr Res. 2014 Jan;152(1):289-94. doi: 10.1016/j.schres.2013.11.013. Epub 2013 Dec 11.
6. Stéphanie Grot et al. Converting scores between the PANSS and SAPS/SANS beyond the positive/negative dichotomy. Psychiatry Res. 2021 Nov;305:114199. doi: 10.1016/j.psychres.2021.114199. Epub 2021 Sep 1.
7. K Kroenke, R L Spitzer, J B Williams. The PHQ-9: validity of a brief depression severity measure. J Gen Intern Med. 2001 Sep;16(9):606-13. doi: 10.1046/j.1525-1497.2001.016009606.x.PMID: 11556941

**Table S1:** Additional information of the included randomized controlled trials.

| **Author**  **Year** | **Patients’ characteristics** | | | | | | | | | **Intervention** | **Antipsychotics** | | | | **Other concomitant treatments** | |
| --- | --- | --- | --- | --- | --- | --- | --- | --- | --- | --- | --- | --- | --- | --- | --- | --- |
|  | **N** | **Males [n(%)]** | **u18** | **age at diagnosis**  **[yrs]** | **Disease**  **Dur [yrs]** | **age [yrs]** | **Stable disease** | **DM** | **BMI at baseline**  **(Kg/m^2^)** | **Type &**  **Dose**  **(mg/die)** | **Type [n(%)]**  **If more than one AP, the most used is in bold.** | **naïve** | **Dur [yrs]** | **Fixed**  **dose** | **Life**  **style** | **Others [n(%)]** |
| Agarwal 2021 | 21 | 12 (57.1) | Y | 23.8 (7.66) | 7.53 (6.16) | 31.4 (6.51) | Y | Y | 38.8 (15.1) | Metformin  (1000-1500) | ARI: 1 (4.76)  **CLO: 5 (23.8)**  FLU: 1 (4.76) OLA: 2 (9.52)  PAL-P: 1 (4.76)  PERPH: 1 (4.76) QUE: 3 (14.3) RIS: 2 (9.52)  ZIP: 1 (4.76) ZUCL: 1 (4.76) Poly: 3 (14.3) | N | - | - | Y | - |
|  | 9 | 2 (22.2) |  | 21.3 (4.90) | 10.94 (7.58) | 32.2 (6.14) |  |  | 42.4 (9.86) | Placebo | **ARI: 4 (44.4)**  CLO: 1 (11.1)  PAL: 1 (11.1) RIS-in: 1 (11.1) Poly: 2 (22.2) |  |  |  |  |  |
| Baptista 2006 | 19 | 10 (52.6) | - | - | - | M: 47.9 (10.6) F: 47.4 (5.9) | Y | N | 23.1 (2.8) | Metformin  (850-1750) | OLA+FLUPH-dec or HAL: 19(100) | N | 30.7 (10.1) | Y | Y | - |
|  | 18 | 12 (66.7) |  |  |  |  |  |  | 23.0 (3.3) | Placebo | OLA+FLUPH-dec or HAL:  18 (100) |  |  |  |  |  |
| Baptista 2007 | 36 | 23 (63.9) | N | - | - | M: 42.4 (11.7) F: 46.2 (11.3) | - | N | 25.0 (4.9) | Metformin  (850-2550, adjusted to individual tolerance) | OLA: 36 (100) | N | OLA: 6,7 (10,8) months | N | N | - |
|  | 36 | 19 (52.8) |  |  |  | M: 43.2 (14.5) F: 46.0 (9.1) |  |  | 26.18 (5.7) | Placebo | OLA: 36 (100) |  |  |  |  |  |
| Carrizo 2009 | 31 | 20 (83.3) | N | - | - | 39.6 (9.7) | - | N | 28.7 (5.3) | Metformin  (1000) | CLO: 31 (100) | N | 8.2 (3.4) | N | Y | - |
|  | 30 | 23 (76.7) |  |  |  | 38.3 (8.7) |  |  | 27.4 (5.7) | Placebo | CLO: 30 (100) |  | 6.6 (3.3) |  |  |  |
| Chen 2013 | 28 | 13 (46.4) | N | 21.2 (5.8) | - | 41.8 (7.2) | - | N | 25.9 (3.9) | Metformin  (1500) | CLO: 28 (100) | N | 5.7 (4.5) | Y | N | - |
|  | 27 | 15 (55.6) |  | 20.8 (5.3) |  | 41.4 (10.2) |  |  | 25.7 (4.3) | Placebo | CLO: 27 (100) |  | 5.1 (4.0) |  |  |  |
| Chiu  2016 | 18 | 8 (44.4) | N | 21.7 (5.9) | 21.4 (8.5) | 42.1 (8.4) | - | N | 24.6 (3.5) | Metformin (500) | CLO: 18 (100) | N | - | Y | N | - |
|  | 19 | 8 (42.1) |  | 20.8 (5.6) | 27.8 (6.0) | 50.3 (6.1) |  |  | 26.6 (3.2) | Metformin (1000) | CLO: 19 (100) |  |  |  |  |  |
|  | 18 | 8 (44.4) |  | 20.8 (6.5) | 25.1 (9.7) | 44.2 (10.0) |  |  | 26.7 (3.8) | Placebo | CLO: 18 (100) |  |  |  |  |  |
| Hebrani  2015 | 30 | 10 (55) | N | - | 21.1 (9.2) | 47.2 (10.4) | - | N | 30.2 (3.3) | Metformin (1000) | CLO: 30 (100) | N | 0.42 (0.42) | N | N | - |
|  | 30 | 7 (41.5) |  |  | 19.3 (10.2) | 45.8 (10.2) |  |  | 28.8 (4.7) | Placebo | CLO: 30 (100) |  | 0.59 (0.51) |  |  |  |
| Mondal 2014 | 41 | - | - | - | - | - | - | - | - | Control | OLA: 41 (100) |  | - | Y (OLA 5 - 20 mg/die) | - | - |
|  | 41 |  |  |  |  |  |  |  |  | Metformin (1000) | OLA: 41 (100) |  |  |  |  |  |
|  | 41 |  |  |  |  |  |  |  |  | Topiramate  (100) | OLA: 41 (100) |  |  |  |  |  |
| Siskind 2021 | 10 | 7 (70) | N | - | - | 36.2 (13.8) | - | N | 25.5 (1.5) | Metformin  (2000) | CLO: 10 (100) | Y | - | N | Y | - |
|  | 10 | 10 (100) |  |  |  | 30.7 (7.7) |  |  | 29.9 (4.3) | Placebo | CLO: 10 (100) |  |  |  |  |  |
| Tang  2021 | 8 | 4(50) | Y | - | - | 25.0 (3.9) | Y | N | 27.9 (6.4) | Metformin  (1201.2) | AMI: 1 (12.5)  ARI: 1 (12.5)  FLU: 1 (12.5)  PAL: 2 (25.0)  **RIS: 3 (37.5)** | N | - | N | N | Anticholin:  1 (12.5) AD:  2 (25) Mood st:  2 (25) |
|  | 9 | 5(55.6) |  |  |  | 24.0 (6.0) |  |  | 30.7 (4.8) | Placebo | ARI: 2 (22.2)  CLO: 1 (11.1)  FLU: 2 (22.2) OLA: 1 (11.1)  PAL: 1 (11.1) **RIS: 3 (33.3)** |  |  |  |  | AD:  3 (33.3) Mood st:  1 (11.1) BDZ 1(11.1) |
| Wang 2012 | 32 | 15 (47) | N | - | 9 (2.6) months | 26.8 (4.2) | Y | N | 24.7 (1.0) | Metformin  (1000) | **CLO: 10 (31.2)**  SUL: 6 (18.8) RIS: 8 (25.0) OLA: 8 (25.0) | N | 7.7 (2.8) months | Y | N | - |
|  | 34 | 19 (56) |  |  | 9.1 (2.3) months | 25.6 (4.6) |  |  | 24.3 (1.2) | Placebo | **CLO: 11 (32.4)**  OLA: 7 (20.6)  RIS: 8 (23.5)  SUL: 8 (23.5) |  | 7.8 (2.2) months |  |  |  |
| Wu  2008a | 32 | 16 (50) | N | - | 9.3  (8.4-10.2)* months | 26.8  (25.2-28.3)* | Y | N | 24.6 (24.2-25.1)* | Metformin  (750) | **CLO: 11 (34.4)** OLA: 8 (25.0), RIS: 7 (21.9),  SUL: 6 (18.8) | N | 8.1 (7.2-8.9)* months | Y | N | Trihexyphenidyl or  Lorazepam  as needed |
|  | 32 | 16 (50) |  |  | 8.9  (8.1-9.8)* months | 25.8  (24.1-27.6)* |  |  | 24.5 (24.2-24.9)* | Placebo | **CLO: 10 (31.3)** OLA: 7 (21.9) RIS: 8 (25.0) SUL: 7 (21.9) |  | 7.7 (6.8-8.5)* months |  |  |  |
|  | 32 | 15 (46.9) |  |  | 8.9 (7.8-10.0)* months | 26.1  (24.3-27.8)* |  |  | 24.6 (24.1-25.0)* | Metformin  (750) | **CLO: 10 (31.3)** OLA: 8 (25.0) RIS: 7 (21.9) SUL: 7 (21.9) |  | 7.6 (6.5-8.6)* months |  | Y |  |
|  | 32 | 17 (53.1) |  |  | 9 (8.0-10.0)* months | 26.4  (24.8-28.1)* |  |  | 24.6 (24.1-24.9)* | Placebo | CLO: 9 (28.1)  **OLA: 10 (31.3)** RIS: 6 (18.8) SUL: 7 (21.9) |  | 7.7 (6.7-8.7)* months |  |  |  |
| Wu  2008b | 20 | 10 (55.6) | N | 25.4 (3.9) | 6,8 (3,1) months | 25.4 (3.9) | N | N | 21.32 (0.56) | Metformin  (750) | OLA: 18 (100) | Y | - | Y (OLA 15 mg/die) | Y | - |
|  | 20 | 10 (52.6) |  | 24.8 (3.5) | 7,6 (3,6) months | 24.8 (3.5) |  |  | 21.57 (0.62) | Placebo | OLA: 19 (100) |  |  |  |  |  |
| Wu  2016 | 103 | - | N | 26.27 (4.53) | 9.05 (2.69) months | 26.27 (4.53) | N | N | 24.57 (1.29) | Metformin  (1000) | CLO: 17 (16.5) **OLA: 51 (49.5)** RIS: 27 (26.2) SUL: 8 (7.8) | Y | - | Y | N | trihexyphenidyl or Lorazepam as needed |
|  | 98 |  |  | 25.74 (4.68) | 8.46 (2.50) months | 25.74 (4.68) |  |  | 24.83 (1.19) | Placebo | CLO: 12 (12.2) **OLA: 45 (45.9)** RIS: 28 (28.6)  SUL: 13 (13.3) |  |  |  |  |  |

If not specified, continuous variables are reported as mean(SD). *Median (IQR).

AMI: amisulpride; AP: antipsychotic; ARI: aripiprazole; CHLO: chlorpromazine; CLO: clozapine; DM: diabetes mellitus; FLU: flupentixol; FLUPH: fluphenazine; FLUPH-dec: fluphenazine decanoate; HAL: haloperidol; IOX: ioxitane; OLA: olanzapine; PAL: paliperidone; PAL-P: paliperidone palmitate; PERPH: Perphenazine; Poly: polytherapy; QUE: quetiapine; RIS: risperidone; RIS-in: risperidone, injectable; SUL: sulpiride; TCA: THIO: thiothixene; ZIP: ziprasidone; ZUCL: Zuclopenthixol.

**Table S2.** Adverse Events (AEs) reported in every single trial

| **Author, year** | **Treatment**  **(n° of patients)** | **General AEs [n (%)]** | **Psychiatric AEs**  **[n (%)]** |
| --- | --- | --- | --- |
| Agarwal, 2021 | Metformin  (21) | Abdominal bloating 3 (14.3); Abdominal pain 2 (9.52); Acid reflux 2 (9.52); Constipation 4 (19.0); Death 1 (4.8); Decreased appetite 2 (9.52); Diarrhoea 8 (38.1); Dizziness 3 (14.3); Dysgeusia 2 (9.52); Fatigue 1 (4.8); Functional dyspepsia 1 (4.8); Headache 1 (4.8); Increased appetite 1 (4.8); Influenza-like illness 1(4.8); Lightheadedness 1(4.8); Muscle spasm 2 (9.52); Myalgia 1 (4.8); Nausea 9 (42.9); Paraesthesia 2 (9.52); Pregnancy (4.8); Pruritus 1 (4.8); Pulmonary Embolism 1 (4.8); Vomiting 3 (14.3); Xerostomia 2 (9.52) | Increase in psychosis 1(4.8);  Irritated/Bad mood 1 (4.8) |
|  | Placebo  (9) | Abdominal pain 1 (11.1); Constipation 4 (44.4); Decreased appetite 2 (22.2); Diarrhoea 7 (77.8); Difficulty concentrating 1 (11.1); Dizziness 3 (33.3); Fatigue 2 (22.2); Functional dyspepsia 2 (22.2); Headache 1 (11.1); Increased appetite 1 (11.1); Influenza-like illness 1 (11.1); Lightheadedness 1 (11.1); Myasthenia 1 (11.1); Nausea 5 (55.6); Palpitations 1(11.1); Rash 1(11.1); Vomiting 2(22.2) | Depression 1 (11.1) |
| Baptista, 2006 | Metformin  (19) | Mild gastrointestinal discomfort | - |
|  | Placebo  (18) | - | - |
| Baptista, 2007 | Metformin  (36) | Mild gastrointestinal discomfort | - |
|  | Placebo  (36) | - | - |
| Carrizo, 2009 | Metformin  (31) | Epigastric uneasiness 1 (4.2) | Unstable disease 6 (19.3) |
|  | Placebo  (30) | - | - |
| Chen, 2013 | Metformin  (28) | Diarrhoea 9 (32.1); Nausea and vomiting 7 (25.0) | - |
|  | Placebo  (27) | Diarrhoea 5 (18.5); Nausea and vomiting 3 (11.1) | - |
| Chiu, 2016 | Metformin 500 mg  (18) | Diarrhoea 2 (11.1); Nausea or vomiting 4 (22.2) | - |
|  | Metformin 1000 mg  (19) | Diarrhoea 5 (26.3); Nausea or vomiting 4 (21.1) | - |
|  | Placebo  (18) | Diarrhoea 5 (27.8); Nausea or vomiting 3 (16.7) | - |
| Hebrani, 2015 | Metformin  (30) | Gastrointestinal discomfort and nausea | - |
|  | Placebo  (30) | - | - |
| Mondal, 2014 | Control (41) | - | - |
|  | Metformin (41) | - | - |
|  | Topiramate (41) | - | - |
| Siskind, 2021 | Metformin  (10) | Abdominal discomfort 1 (10); Constipation 1 **(**10)**;** Diarrhoea 2(20); Dizziness 1(10); Foot drop 1(10); Heartburn 3(30); Hypertension 3(30); Nausea 1(10); Postural Hypotension 1 (10); Tachycardia 6 (60); URTI 2 (20) | - |
|  | Placebo  (10) | Abdominal discomfort 2 (20); Constipation 1 (10); Decreased Appetite 1 (10); Heartburn 1 (10); Hypertension 4 (40); Nausea 1 (10);  Myocarditis 1 (10); Tachycardia 4 (40); URTI 1 (40) | - |
| Tang, 2021 | Metformin  (8) | Diarrhoea 2 (25); Nausea and vomiting 1 (12.5); Soft stools 1 (12.5) | Psychotic relapse 1 (12.5) |
|  | Placebo  (9) | Asthenia 1 (11.1); Diarrhoea 1 (11.1); Dizziness 1 (11.1); Flatulence 2 (22.2); Headache 1 (11.1); Indigestion 1 (11.1); Light-headedness 1 (11.1) Nausea 1 (11.1); Vomiting 2 (22.2); | - |
| Wang, 2012 | Metformin  (32) | Dry mouth 11 (34.4); EPS 11 (34.4); Nausea 9 (28.1) Somnolence 9 (28.1) | Insomnia and agitation 7(21.9); Exacerbation of psychosis 2 (6.3) |
|  | Placebo  (34) | Dry mouth 8 (23.5); EPS 10 (29.4) Nausea 7 (20.6) Somnolence 9 (26.5) | Insomnia and agitation 8 (23.5); Exacerbation of psychosis 1 (2.9) |
| Wu, 2008(a)* | Metformin  (32) | Dry mouth 3 (9.4); EPS 6 (18.8); Headache 3 (9.4); Nausea 6 (18.8); Somnolence 3 (9.4) | Insomnia and agitation 6 (18.8) |
|  | Placebo  (32) | Diabetes 2 (6.3); Dry mouth 2 (6.3); EPS 9 (28.1); Headache 2 (6.3); Nausea 4 (12.5); Somnolence 2 (6.3) | Insomnia and agitation 6(18.8); Exacerbation of psychosis 1 (3.1) |
|  | Metformin +Life style  (32) | Dry mouth 3 (9.4); EPS 8 (25); Headache 3 (9.4); Nausea 4 (12.5); Somnolence 2 (6.3) | Insomnia and agitation 5(15.6); Exacerbation of psychosis 2 (6.3) |
|  | Placebo +Life style  (32) | Dry mouth 2 (6.3); EPS 7 (21.9); Headache 2 (6.3); Nausea 5 (15.6); Somnolence 3 (9.4) | Insomnia and agitation 6(18.8); Exacerbation of psychosis 2 (6.3) |
| Wu, 2008(b)* | Metformin  (20) | Nausea 2 (10.0) | - |
|  | Placebo  (20) | Nausea 2 (10.0) | - |
| Wu, 2016 | Metformin  (103) | Dry mouth 8 (7.8); EPS 21 (20.4); Nausea 23 (22.3); Somnolence 7 (6.8) | Insomnia and agitation 10 (9.7) |
|  | Placebo  (98) | Dry mouth 5 (5.1); EPS 19 (19.4); Nausea 18 (18.4); Somnolence 4 (4.1) | Insomnia and agitation 9 (9.2) |

AE: adverse events; EPS: extrapyramidal symptoms; URTI: upper respiratory tract infections.

*Wu, 2008(a): Wu, R.R., et al. (2008). "Lifestyle intervention and metformin for treatment of antipsychotic-induced weight gain: A randomised controlled trial." JAMA - Journal of the American Medical Association 299(2): 185-193.

Wu, 2008(b): Wu, R.R., et al. (2008). "Metformin addition attenuates olanzapine-induced weight gain in drug-naive first-episode schizophrenia patients: A double-blind, placebo-controlled study." American Journal of Psychiatry 165(3): 352-358.

**Figure S1:** Metformin compared to Placebo considering PANSS


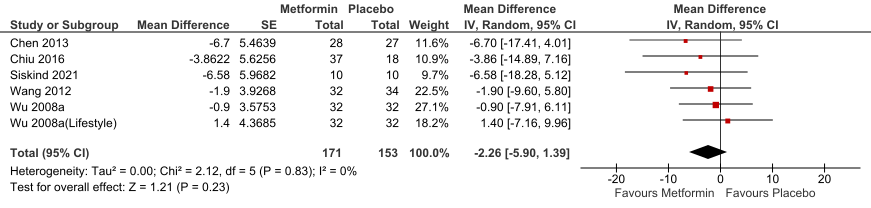


**Figure S2:** Metformin compared to Placebo considering BPRS


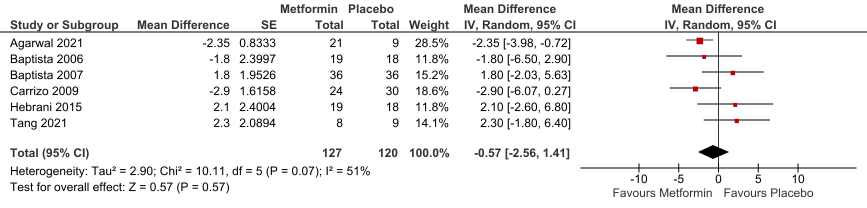


**Figure S3:** Metformin compared to Placebo considering GAF


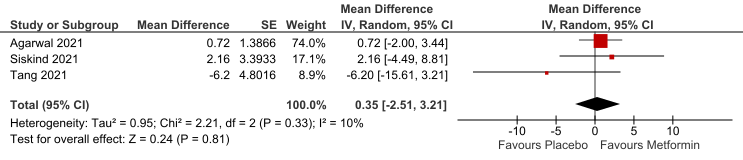

Supplement: Supplementary file 1 [file Data_Sheet_1.docx]
